# Soil Column Experimental Study on the Effect of Soil Structure Disturbance on Water Chemistry

**DOI:** 10.3390/ijerph192315673

**Published:** 2022-11-25

**Authors:** Yin Long, Tianming Huang, Fen Zhang, Yajing Zhao

**Affiliations:** 1 Key Laboratory of Shale Gas and Geoengineering, Institute of Geology and Geophysics, Chinese Academy of Sciences, Beijing 100029, China; 2 College of Earth and Planetary Sciences, University of Chinese Academy of Sciences, Beijing 100049, China

**Keywords:** sulfate, soil column experiment, water–rock interaction, sulfur and oxygen isotopes, water chemistry, loess

## Abstract

The changes in soil/rock structure caused by engineering disturbance or earthquakes could affect water chemistry by increasing the reaction surface, enhancing the oxidation condition, or exposing soluble rocks. However, the details of the mechanisms of the disturbance of soil/rock are little known. Based on the soil column experiment, this study analyzed the concentrations of sulfate (SO_4_), sulfur, and oxygen isotopic composition of SO_4_ (δ^34^S-SO_4_ and δ^18^O-SO_4_) in effluent water. The water–rock interaction mechanisms in the disturbed soil and the contribution of this interaction to the SO_4_ in groundwater were studied. The results suggest that the concentration of SO_4_ in the first effluent water sample can reach up to 97 mg/L, much higher than that in natural groundwater (6.8 mg/L). The isotopic composition of SO_4_ further suggested that SO_4_ in the first effluent water sample was mainly derived from the dissolution of SO_4_-containing evaporites. The proportion was estimated to be 93%. SO_4_-containing evaporites accounted for 23% of the SO_4_ content in all effluent water samples during the experiment. The disturbance of soil structure led to the exposure and dissolution of SO_4_-containing evaporites, which were initially insoluble under natural conditions. This study is essential to the clarification of the water–rock interaction mechanisms following the changes in soil/rock structures.

## 1. Introduction

The unsaturated zone is integral to the Earth’s critical zone, connecting the surface and groundwater. It plays an indispensable role in the process of groundwater recharge from precipitation. As a result of societal progress, infrastructure engineering is increasingly being more widely implemented. Examples include China’s campaign to bulldoze mountains to build cities, resulting in the bulldozing of the top of loess mountains, and the filling of gullies to create land for cities [1]. These soil disturbances due to engineering could destroy the natural structure of the soil [2] and affect water quality [3,4,5]. Previous studies have found that the changes in water chemistry following earthquakes are related to the minerals being exposed to fluids [6,7]. Changes in water chemistry may also be due to the change in redox conditions [8]. In addition, when water flows through the reactive fine-grained material [9], it could act as a solute generator affecting water quality [10,11]. At the same time, the flow path of water is an essential factor affecting the solute flux change caused by water–rock interactions [12,13]. When the flow path changes, the solute flux also changes [6,13]. In disturbed conditions, soil disturbance can damage the soil structure [14], expose minerals, and change the water flow path [6]. Therefore, the mechanisms of the water–rock interaction are the key to understanding the influence of water–rock interactions on water chemistry and the evolution of solutes.

Sulfate (SO_4_)-containing evaporites are common soluble salts in soil [15]. Concerning the effect of soil structural damage on water chemistry, SO_4_ can vary significantly. Therefore, tracing the source of SO_4_ is useful in clarifying the mechanism of its impact on water chemistry. In recent years, many studies have used isotopes to trace water–rock interactions and the origins of solutes in groundwater under natural conditions [16,17,18]. The SO_4_ in groundwater can be controlled by atmospheric deposition and the water–rock interaction [19,20,21,22,23]. In terms of the impact of disturbed soil on the environment, many studies have focused on the impact of changes in contaminants transport (organic matter, heavy metals, etc.) on water quality after soil structure destruction [24,25,26]. However, it has been proved that soil disturbance can significantly affect the major ions in the water environment [13]. Previous studies have analyzed the influence of disturbed soil on the SO_4_ content in groundwater through water–rock interactions, such as SO_4_-containing evaporite dissolution and pyrite oxidation [16,27]. However, the detailed causes and mechanisms of water–rock interaction have not been discussed. How does the dissolution reaction occur, and in how many ways do minerals dissolve? Currently, existing studies on the mechanisms of the water–rock interaction in disturbed soil are insufficient. The alteration of the disturbed soil to groundwater solutes has not been well quantified, and there is no clear distinction between the solutes coming directly from primary minerals and those from other sources [6]. Therefore, a knowledge gap exists in the details of water–rock interactions following the disturbance of soils.

Loess is a typical structural soil and soluble salt is a critical component supporting its structure [15]. The soil structure changes significantly when the loess is disturbed [28]. Therefore, loess is an ideal soil to study the impact of soil disturbance on water quality. Under natural conditions, SO_4_ in the groundwater (all were premodern and old water) of the Xifeng Loess Tableland in the Loess Plateau of China was mainly recharged by old precipitation [21,29]. Under disturbance conditions, the soil structure changes. When the precipitation percolates through the unsaturated zone, the water–rock interactions that are dominated by leaching may change. In this study, a soil column experiment with disturbed soil was conducted. It aimed to clarify the origins of SO_4_ in the effluent water using isotopic methods (δ^34^S-SO_4_ and δ^18^O-SO_4_) and compare the effluent water with the percolating groundwater under natural conditions to illustrate the impact of soil disturbance on water chemistry. This study provides insights into the detailed mechanisms of the water–rock interaction following the disturbance of loess samples.

## 2. Materials and Methods

A soil column experiment was designed to investigate the response of soil disturbance to the concentrations of SO_4_ in effluent water. Loess, a typical structural soil, was used in the experiment. This experiment used a control variable: soil remolding, which is regarded as a disturbance. The processing of sampling, mixing, and filling, to some extent, represented disturbance to the soil during engineering, such as excavation and compaction of loess. The δ^34^S-SO_4_ and δ^18^O-SO_4_ can quantitatively trace the sources of SO_4_ in the effluent water. The effect of soil disturbance on water chemistry was illustrated by comparing the effluent water of the soil column with the percolating groundwater under natural conditions. A combination of the soil column experiment and isotope method quantitatively described the sources and changes of SO_4_ in the whole process of water percolation through the disturbed soil.

### 2.1. Mineral Composition Analysis

X-ray diffraction (XRD) analysis is usually used to determine the main mineral composition in soil. However, when the sulfate content (C, ppm) in the soil is too small to be determined by XRD analysis, deionized water can be used to extract sulfate to represent SO_4_ in the soil. According to the law of conservation of mass, the formula is as follows:

C = C_e_·V/M (1)

where C_e_ (mg/L) is the SO_4_ concentration of the supernatant solution, V (mL) is the volume of deionized water added into the oven-dried soil, and M (g) is the mass of the extracted oven-dried soil. We can also obtain the water-extractable concentration (C_s_) by the following formulas:

C_e_·V = M·θ_g_/ρ_w_·C_s_ (2)

C_s_ = C_e_·V·ρ_w_/M/θ_g_ (3)

where θ_g_ (%) is the gravimetric moisture content and ρ_w_ is the density of water, at 1 g/mL. First, three loess samples with different depths from soil profile XZ1 in the Xifeng Loess Tableland were chosen to analyze mineral compositions. If SO_4_-containing minerals were not detected, then deionized water extraction experiments were conducted. 50 mL of deionized water was mixed with 50 g of oven-dried soil. The water-extractable concentration of SO_4_ (C_s_) was measured from the supernatant solution. The natural soil θ_g_ was determined by drying a minimum of 80 g of soil at 105 °C for 24 h. 

### 2.2. Soil and Groundwater in Natural Conditions

The soil profile XZ1, with a depth of 55 m drilled on the Xifeng loess platform, reaches a groundwater level of 54 m and penetrates the aquifer within 1 m [29]. The water-extractable concentration of SO_4_ in the soil profile XZ1 showed that SO_4_ in the shallow unsaturated zone (<10 m) was mainly derived from anthropogenic sources and the dissolution of evaporite minerals, while SO_4_ in the deep unsaturated zone (>10 m) was mainly derived from the dissolution of evaporite minerals [21]. In order to ensure that the experiment contained no other variables than the control variables (nature and disturbance), the soil used to fill the soil column was from the natural loess with a depth of 42.5–53 m in the soil profile XZ1. The characteristics of evaporite-containing SO_4_ in soil with a depth of 42.5–53 mare represented by L0.

The groundwater represents the water percolation through the soil profile (i.e., unsaturated zone) under natural conditions [29]. Therefore, the natural groundwater was in the initial state compared with the effluent water percolating through the disturbed soil. The influent water in the soil column experiment was the groundwater from the soil profile XZ1, and G0 represents the characteristics of groundwater.

### 2.3. The Soil Column Experiment

**Figure 1:**
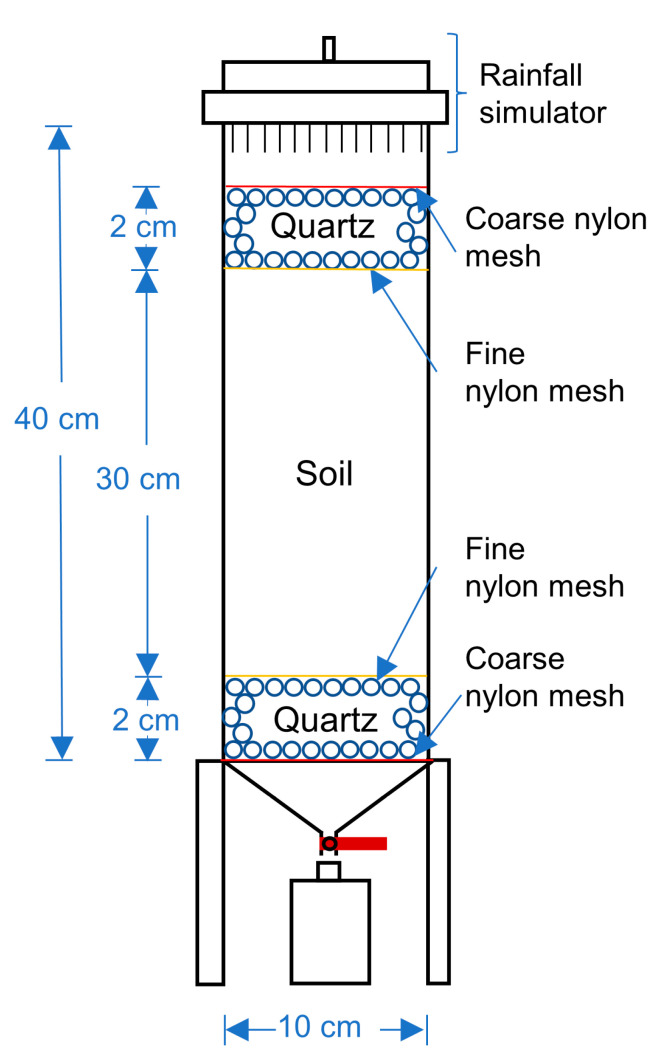
Schematic diagram of soil column structure.

The internal diameter of the soil column was 10 cm. The height was 40 cm (Figure 1). From bottom to top, the filling materials in the soil column were coarse nylon mesh, 2-cm-depth quartz sand, fine nylon mesh, 30 cm soil column, fine nylon mesh, 2 cm quartz sand, coarse nylon mesh, and a rainfall simulator. The dried soil was filled and compacted in layers with a density of 1.5 g/cm^3^ (i.e., 117.75 g loess samples per cm in the column). A total of 30 cm of loess was filled with a total mass (M) of 3532.5 g.

The influent water flowed into the packed soil column through the rainfall simulator. The inlet flow rate was adjusted to about 1–3 mL/min by Markov bottle. There was no ponding on the soil surface. The water percolated in the form of an unsaturated flow. The experiment lasted 28 days. The effluent water samples were collected at different time intervals. The experiment was terminated when there was no effluent water coming out of the soil column for three days since the inflow had stopped. About 170 g of soil samples from the upper, middle, and lower parts of the soil column with an interval of 10 cm were taken. The water contents were measured by drying a minimum of 170 g of soil at 105 °C for 24 h. The average value of the three parts of θ_g_ and C_s_ was the soil column’s residual gravimetric moisture content and remaining water-extractable soil water (R1).

### 2.4. Sample Analyses

The mineral composition was analyzed using XRD (X’Pert Pro, Malvern Panalytical Ltd., Malvern, U.K.). The concentrations of SO_4_ and Cl were analyzed using Ion Chromatography (Dionex ICS-1100). The precision of measurement was 3%. The δ^34^S-SO_4_ values were analyzed using Delta V Plus isotope ratio mass spectrometry (IRMS), and the δ^18^O-SO_4_ values were analyzed using MAT-253 IRMS in the laboratories of the Beijing Research Institute of Uranium Geology. The BaSO_4_ samples were decomposed at 1100 °C with S converted to SO_2_. Subsequently, SO_2_ was introduced to the mass spectrometer’s ion source, and δ^34^S-SO_4_ values were determined. For measuring the ^18^O/^16^O ratio, the BaSO_4_ samples were decomposed with O converted to CO at temperatures of 1350 °C. Following this, the CO gas was swept by a carrier gas into the mass spectrometer, and δ^18^O values were determined. The values of δ^34^S-SO_4_ and δ^18^O-SO_4_ were normalized to the Vienna-Canyon Diablo troilite (VCDT) and Vienna Standard Mean Ocean Water (VSMOW) reference standards, respectively. The precision of the δ^34^S-SO_4_ and δ^18^O-SO_4_ measurements was 0.2‰ and 0.2‰, respectively.

## 3. Results

### 3.1. Mineral Composition of the Soil

The results of the XRD are listed in Table 1. The main minerals in the soil were quartz and clay, followed by plagioclase, calcite, and potassium feldspar. The illite in the clay minerals was the highest. However, the SO_4_-containing evaporites were not detected. Based on the water-extraction methods (Formula (1)), the average concentration of SO_4_ in the soil-column sample from a depth of 42.5–53 m (L0) was 23 ppm. The average values of δ^34^S-SO_4_ and δ^18^O-SO_4_ were 14.1‰ and 9.1‰, respectively, which belong to the SO_4_-containing evaporites within the δ^34^S-SO_4_ value range of 10–35‰ [30].

### 3.2. SO_4_ in Soil and Groundwater under the Natural Conditions

The results of δ^34^S-SO_4_, δ^18^O-SO_4_, and SO_4_ are listed in Table 2. The weight-weighted average of θ_g_ of natural soil at a depth of 42.5–53 m was 22.6%.

The influent water in the column experiment was a mixture of several groundwater samples (the variation of water chemistry and isotopic compositions was small) reported in our other study [21]. The concentrations of SO_4_ and Cl were 6.8 mg/L and 5.5 mg/L, respectively. The δ^34^S-SO_4_ and δ^18^O-SO_4_ values of the influent water were 7.2‰ and −2.3‰, respectively (Table 2).

### 3.3. The Soil Column Experiment

In the initial soil column, the total amount of Cl was estimated to be 4.4 mg (3532.5 g × 22.6% × 5.5 mg/L, when the concentration of 5.5 mg/L of influent water was considered the concentration of actual soil water). The amount of influent water (G0) was 40.1 L. After the experiment, the average value of θ_g_ was 30.5%. When 220.6 mg (5.5 mg/L × 40.1 L) of Cl had flowed into the soil column, the content of Cl in the effluent water and the remaining Cl in the soil column was 217.7 mg (Table 2, sum(Cl × V)) and 3.9 mg (3532.5 g × 30.5% × 3.6 mg/L), respectively. According to the mass balance, the sum of the content of Cl in the influent water and the initial soil was 225.0 mg (220.6 + 4.4 mg), while the sum of Cl in the effluent water and the residual soil column was 221.6 mg (217.7 + 3.9 mg). The difference before and after the experiment was tiny at 3.4 mg, and the mass unbalance error was 1.5% (3.4/225.0 × 100%). Therefore, Cl was stable in the soil column system, and there was an absence of Cl-minerals (e.g., halite) in the soil; the value of SO_4_/Cl can assist in tracing the sources of SO_4_.

**Figure 2:**
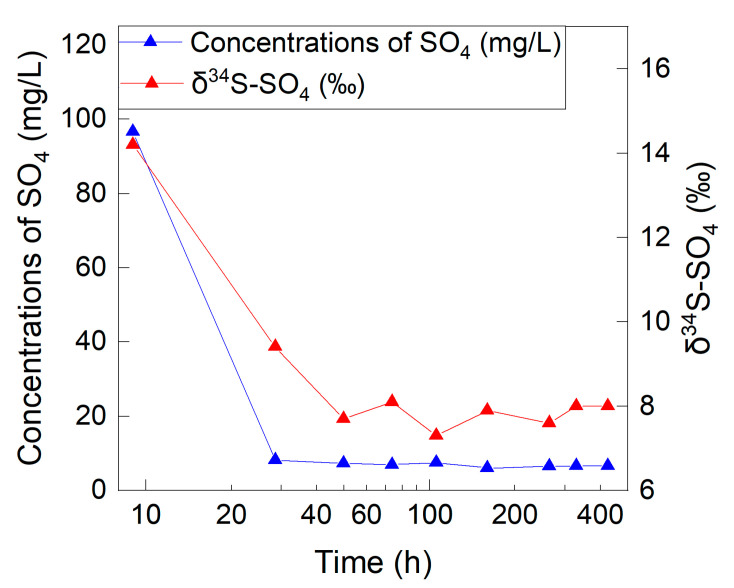
Change in concentrations of SO_4_ and δ^34^S-SO_4_ in the effluent water over time.

The concentrations of SO_4_ in the effluent water collected from the soil column ranged from 6.0 mg/L to 96.6 mg/L. The concentrations of SO_4_ decreased sharply within 30 h of percolation and tended to be stable after 50 h of leaching (Figure 2). The concentration of SO_4_ was 96.6 mg/L for the first effluent water sample within 7 h (L1), and it was 6.6 mg/L for the last effluent water sample (L9), very close to the influent water sample (6.8 mg/L). The SO_4_/Cl mass ratio of the first effluent water sample (8.6) was about seven times higher than that of the final effluent water sample (1.2). This indicated that SO_4_ from other sources besides the influent water was leached out. As the concentration of SO_4_ in the actual soil water in the deep unsaturated zone was consistent with that in the groundwater, the SO_4_ content from the soil water after drying in the initial soil column was 5.4 mg (3532.5 g × 22.6% × 6.8 mg/L). When 272.7 mg (6.8 mg/L × 40.1 L) of SO_4_ flowed into the soil column, the content of SO_4_ in the effluent water and the remaining SO_4_ in the soil column was 350.5 mg (Table 2, sum(SO_4_ × V)) and 9.6 mg (3532.5 g × 30.5% × 8.9 mg/L), respectively. Among the remaining 9.6 mg of SO_4_, 7.3 mg (3532.5 g × 30.5% × 6.8 mg/L) were from the influent water, and 2.3 mg were from other sources. According to the mass balance, the sum of the content of SO_4_ in the influent water and the initial soil was 278.1 mg (272.7 + 5.4 mg), while the sum of the content of SO_4_ in the effluent water and the residual soil column was 357.8 mg (350.5 + 7.3 mg). The difference before and after the experiment was 79.7 mg, suggesting that 79.7 mg of dissolved SO_4_ originated from other sources.

**Figure 3:**
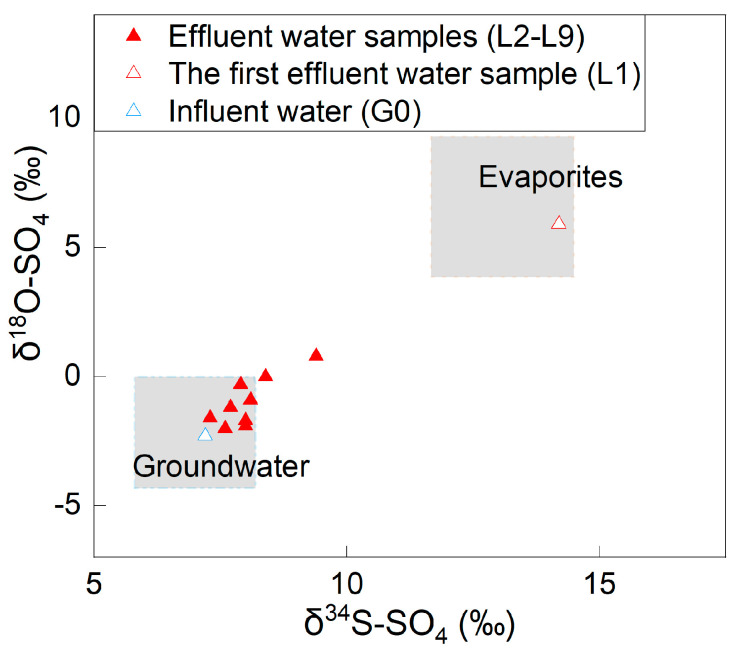
Sulfur and oxygen isotope distribution in the effluent water (the ranges of the δ^34^S-SO_4_ and δ^18^O-SO_4_ values of SO_4_-containing evaporites and groundwater were adopted from Long et al. [21].

The δ^34^S-SO_4_ values of the effluent water samples ranged from 7.3‰ to 14.2‰, while the δ^18^O-SO_4_ values ranged from −2.0‰ to 5.9‰ (Figure 3). The δ^34^S-SO_4_ changed from 14.2‰ to 8.0‰, and the δ^18^O-SO_4_ changed from 5.9‰ to −1.9‰ from the beginning to the end of the experiment.

## 4. Discussion

### 4.1. SO_4_-Increasing Process in Effluent Water

In our previous study [21], pyrite minerals were not detected in the loess samples, and the oxidation of pyrite minerals that could increase the concentration of SO_4_ was absent. Under the experimental conditions, the aerobic environment in the soil column was not suitable for the SO_4_ reduction reaction [30]. In addition, the water retention time in the soil column was short such that the exchange of sulfur isotope was difficult. Therefore, no redox reaction changed the sulfur and oxygen isotopes significantly. The isotopic characteristics in the effluent water represented the isotopic characteristics of the SO_4_ source.

There were two endmembers of SO_4_ in the soil column system: (1) the influent water (G0) with a δ^34^S-SO_4_ value of 7.2‰, an δ^18^O-SO_4_ value of −2.3‰, and the concentration of SO_4_ of 6.8 mg/L; and (2) the SO_4_-containing evaporites (L0). The average value of δ^34^S-SO_4_ was 14.1‰, and δ^18^O-SO_4_ was 9.1‰ for L0.

On the one hand, the sum of the SO_4_ content in the effluent water and the remaining SO_4_ content in the soil column was greater than that of the influent water plus soil water (357.8 mg > 278.1 mg), indicating that there were additional SO_4_ sources that increased the SO_4_ content in the effluent water. The additional SO_4_ source was the SO_4_-containing evaporites according to the endmembers of SO_4_ in the soil column system. On the other hand, the δ^34^S-SO_4_ and δ^18^O-SO_4_ in the initial stage of the experiment were close to the evaporite endmember, showing that the SO_4_ in the first effluent water mainly originated from SO_4_-containing evaporites (such as gypsum). As the experiment continued, the characteristics of δ^34^S-SO_4_ and δ^18^O-SO_4_ of the effluent water gradually evolved from SO_4_-containing evaporites to influent water (Figure 3). At the later stage of the experiment, the concentrations of SO_4_ in the effluent water underwent small changes. The SO_4_ mainly originated from influent water. The concentrations of SO_4_, δ^34^S-SO_4_, and δ^18^O-SO_4_ indicated that the soil disturbance makes the water dissolve the undissolved SO_4_-containing evaporites stored in the soil under natural conditions. After the experiment finished, there was a tiny amount (2.3 mg) of undissolved SO_4_-containing evaporite residue in the soil.

During the initial stage of the soil column experiment, the SO_4_ in the effluent water mainly originated from SO_4_-containing evaporites according to the δ^34^S-SO_4_. In the first effluent water sample of 9 h, 93% ((L1 − G0)/L1 × 100%) of the SO_4_ came from SO_4_-containing evaporites according to the concentration of SO_4_. During the experiment, the proportion of SO_4_ originating from SO_4_-containing evaporites in the effluent water decreased (Figure 2). At the end of the experiment, the proportion of SO_4_-containing evaporites was 12% ((L9 − G0)/(L0 − G0)) according to δ^34^S-SO_4_. The total content of SO_4_ in the soil column was 82.0 mg (79.7 + 2.3 mg), indicating that the column experiment dissolved 97% (79.7/82.0 × 100%) of SO_4_-containing evaporites when the soil was disturbed. The SO_4_ obtained from SO_4_-containing evaporites accounted for 23% (79.7/350.5 × 100%) of the SO_4_ content in the effluent water, and 77% of the SO_4_ came from the influent water.

### 4.2. Soil SO_4_-Containing Evaporites Dissolution Mechanism

**Figure 4:**
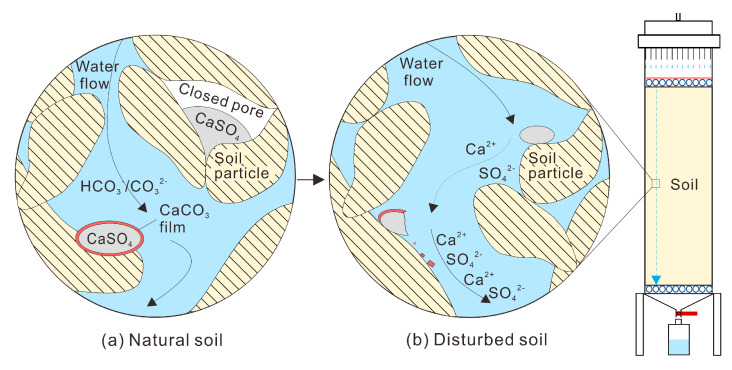
Conceptual diagram of the occurrence of SO_4_-containing evaporites and rock-water interaction mechanisms in the disturbed soil; (**a**) the location of SO_4_-containing evaporites which are not dissolved by water flow in the natural soil; and (**b**) SO_4_-containing evaporites’ dissolution in the disturbed soil.

According to changes in the SO_4_ concentration, δ^34^S-SO_4_, and δ^18^O-SO_4_, disturbance of soil significantly impacts water chemistry compared with soil under natural conditions. The differences in soil structure between disturbed and natural soil mainly concern particle morphology and contact relationships [31]. There is a strong cementation between the particles in the natural loess. The SO_4_-containing evaporites have a cementation and solidification effect on the maintenance of the structure of loess [31]. When the soil was disturbed in the column experiment, most of the original cemented contact was destroyed [32], damaging pores. In this circumstance, the SO_4_-containing evaporites in the cement and the SO_4_-containing evaporites in the closed pores were exposed to the water and dissolved (Figure 4).

Under natural conditions, water moves in an unsaturated flow. Although the water in the soil column had an unsaturated flow, the simulated rainfall generated by the rain simulator percolated in different flow paths. In the process of percolation, unsaturated water can bypass the larger pore channel and form a layer of water film on the surface of soil particles. With the continuous thickening of the water film, the gas in the soil would be trapped in the pores under natural conditions [33]. Under experimental conditions, the soil was disturbed, and the particle morphology and pore structure were changed. Consequently, the pore water and gas adsorption equilibrium in the soil was broken [34]. Furthermore, the transport paths of water, solute, and suspended colloid were changed [35], which accelerated the connection of the water phase in soil pores and the escape process of the originally connected gas phase [34]. Following this, the released percolation channel was occupied by water again. At this time, SO_4_-containing evaporites that were not on the water migration path under natural conditions would have come into contact and dissolved with the water under the new migration path conditions (Figure 4). Some gases that cannot be expelled appear unconnected and are called closed bubbles [34]. The residual SO_4_-containing evaporites in the column may be wrapped by closed bubbles or calcium carbonate film [21]. When SO_4_-containing evaporites exist in some undamaged closed pores, the dissolution of some SO_4_-containing evaporites can also be hindered.

The above two mechanisms are that (1) minerals are exposed to fluid; and (2) the disturbance breaks the water-air balance and generates a new water flow path to increase SO_4_ in the water environment, consistent with the mechanism of solute increase in the water environment found in previous studies [6,7]. Because we did not observe the carbonate film and the sulfate in the closed pores by microscope directly, the mechanisms are as would be expected for such sources and changes. Although we could not distinguish the specific mechanism of dissolution directly, the findings are of significance to the study of the specific mechanism of dissolution.

### 4.3. Implications for Chemical Compositions

The results of the soil column experiment show that soil disturbance had an impact on water chemical compositions. The SO_4_ in the soil can quickly enter the groundwater. Therefore, SO_4_ can be used as one of the parameters to evaluate the impact of disturbance on water chemistry. This is of great significance in evaluating the impact of engineering disturbance on the water environment.

From the perspective of the SO_4_-containing evaporite dissolution mechanisms, the disturbance destroys the soil structure and breaks the original water–gas balance. Although SO_4_ in the effluent water returns to stability after some time, once the soil is disturbed, the SO_4_-containing evaporites on the new water flow path will come into contact with water and dissolve; SO_4_ then enters the groundwater irreversibly. The compaction and other measures taken during the engineering reduce macropores [2], which is conducive to the stability of the project. However, it has no significant effect on preventing the dissolution of evaporites and changing the chemical composition of groundwater. Before the construction of a project, it is necessary to consider the preventive measures for groundwater pollution.

## 5. Conclusions

Isotopes and the soil column experiment provide insight into the potential impact of soil disturbance on SO_4_ concentrations in water. The SO_4_, δ^34^S-SO_4_, and δ^18^O-SO_4_ values of influent water and effluent water samples in the soil column revealed the influence of disturbed soil on water quality. In the natural soil, evaporites exist in closed pores and will not dissolve. When the soil was disturbed, its structure was destroyed. The closed pores and the water–air balance was broken, which changed the migration path of water, and the evaporites dissolved when exposed to the water environment. Therefore, the increase of SO_4_ content in groundwater in a short time was related to the dissolution of SO_4_-containing evaporites in soil. As time went on, the SO_4_ content decreased in the effluent water. At the end of leaching, the SO_4_ content was the same as influent water and was steady because the SO_4_-containing evaporites in the soil were almost entirely dissolved. This study is of great significance for clarifying the mechanisms of the water–rock interaction caused by soil structure damage (such as earthquakes, engineering, etc.). In addition, SO_4_ can be used as one of the evaluation indices used to evaluate the risk of engineering construction to water chemistry.

## Figures and Tables

**Table 1:** The mineral composition for loess (%), modified from [18] under the Creative Commons Attribution (CC BY) license.

Sample	Depth (m)	Quartz	Potassium Feldspar	Plagioclase	Calcite	Dolomite	Clay	Mineral Composition for Clay	
S1	9.75–10	43.0	3.4	16.5	9.1	0	28.0	Smectite	0
								Illite-smectite	32
								Illite	49
								Kaolinite	5
								Chlorite	14
S2	29.5–30	44.7	2.9	11.8	13.6	0	27.0	Smectite	0
								Illite-smectite	20
								Illite	60
								Kaolinite	7
								Chlorite	13
S3	54.5–55	44.5	4.3	15.3	16.4	3.4	16.1	Smectite	0
								Illite-smectite	21
								Illite	58
								Kaolinite	9
								Chlorite	12

**Table 2:** The concentration of SO_4_, δ^34^S-SO_4_, and δ^18^O-SO_4_ values of injected water, remaining water-extractable soil water, and effluent water samples.

Type	Sample No.	Time	Volume	SO_4_	Cl	SO_4_/Cl	δ^34^S-SO_4_	δ^18^O-SO_4_
		(h)	(L)	(mg/L)	(mg/L)		(‰)	(‰)
Influent water	G0		40.1	6.8	5.5	1.2	7.2	−2.3
SO_4_-containing evaporites endmember	L0						14.1	9.1
Effluent water	L1	9	0.98	96.6	11.2	8.6	14.2	5.9
	L2	29	2.02	8.2	7.1	1.2	9.4	0.8
	L3	50	1.97	7.2	5.8	1.2	7.7	−1.2
	L4	74	2.26	6.9	5.1	1.4	8.1	−0.9
	L5	106	3.14	7.5	5.2	1.4	7.3	−1.6
	L6	160	5.23	6.0	5.1	1.2	7.9	−0.3
	L7	264	9.28	6.4	5.3	1.2	7.6	−2.0
	L8	329	6.15	6.6	5.0	1.3	8.0	−1.7
	L9	425	8.21	6.6	5.7	1.2	8.0	−1.9
Remaining water-extractable soil water	R1			8.9	3.6	2.5	*	*

* The SO_4_ content in the solution is below the detection limit.

## Data Availability

Data are contained within the article.
